# Identifying geographical heterogeneity of pulmonary tuberculosis in southern Ethiopia: a method to identify clustering for targeted interventions

**DOI:** 10.1080/16549716.2020.1785737

**Published:** 2020-08-04

**Authors:** Mesay Hailu Dangisso, Daniel Gemechu Datiko, Bernt Lindtjørn

**Affiliations:** a Department of Public Health, College of Medicine and Health Sciences, Hawassa University, Hawassa, Ethiopia; b Department of Clinical Sciences, Liverpool School of Tropical Medicine, Liverpool, UK; c Centre for International Health, Faculty of Medicine, University of Bergen, Bergen, Norway

**Keywords:** Spatial heterogeneity of tuberculosis, Spatial epidemiology, Geographically weighted regression, PTB clustering, Sidama, Ethiopia

## Abstract

**Background:**

Previous studies from Ethiopia detected disease clustering using broader geographic settings, but limited information exists on the spatial distribution of the disease using residential locations. An assessment of predictors of spatial variations of TB at community level could fill the knowledge gaps, and helps in devising tailored interventions to improve TB control.

**Objective:**

To assess the pattern of spatial distribution of pulmonary tuberculosis (PTB) based on geographic locations of individual cases in the Dale district and Yirga Alem town in southern Ethiopia.

**Methods:**

The socio-demographic characteristics of PTB cases were collected using a structured questionnaire, and spatial information was collected using geographic position systems. We carried out Getis and Ord (Gi*) statistics and scan statistics to explore the pattern of spatial clusters of PTB cases, and geographically weighted regression (GWR) was used to assess the spatial heterogeneities in relationship between predictor variables and PTB case notification rates (CNRs).

**Results:**

The distribution of PTB varied by enumeration areas within the kebeles, and we identified areas with significant hotspots in various areas ineach year. In GWR analysis, the disease distribution showed a geographic heterogeneity (non-stationarity) in relation to physical access (distance to TB control facilities) and population density (AICc = 5591, R^2^ = 0.3359, adjusted R^2^ = 0.2671). The model explained 27% of the variability in PTB CNRs (local R^2^ ranged from 0.0002–0.4248 between enumeration areas). The GWR analysis showed that areas with high PTB CNRs had better physical accessibility to TB control facilities and high population density. The effect of physical access on PTB CNRs changed after the coverage of TB control facilities was improved.

**Conclusion:**

We report a varying distribution of PTB in small and different areas over 10 years. Spatial and temporal analysis of disease distribution can be used to identify areas with a high burden of disease and predictors of clustering, which helps in making policy decisions and devising targeted interventions.

## Background

Tuberculosis (TB) is an infectious disease of public health importance causing considerable mortality and economic burden [1]. The burden of the disease varies between and within countries due to a varying distribution of individual and community-level risk factors, and variations in TB control programme performance. Different studies report spatial [2–4] and spatiotemporal clustering [4–8] of the disease. Adverse socio-economic conditions [9–13], a high prevalence of HIV infection [14–17], geographic factors such as altitude [18–21] and access to TB control facilities are all related to variations in the burden of disease.

Ethiopia has been implementing community-based TB control interventions [22], and the country has expanded directly observed treatment short course (DOTS) services to improve access to underserved communities in rural and hard-to-reach areas. As a result, the performance of TB control services has improved; however, the burden of disease remains a public health challenge. An assessment of trends in case notification rates (CNRs) of TB [23], could help understand the performance of TB control programmes such as estimating case detection, and missed cases (who were either not diagnosed and treated or diagnosed but not reported) [24]. Evidence shows that the variations in disease distribution can be due to poor access to diagnostic and treatment facilities [25], or disproportionate distribution of risk factors. Reports from Ethiopia highlighted variations in CNRs [23] and spatiotemporal clustering of TB [26,27]. The variations indicate the importance of targeted interventions and resource allocation at local level based on the burden of the disease.

Spatial analysis of TB helps understand the spatial epidemiology of the disease occurrence in different geographic or administrative levels [25,27]. Understanding factors contributing to the geographic heterogeneities in disease occurrence helps in devising targeted intervention at local level. Evidence from other countries also suggests the importance of understanding the spatial distribution pattern of TB in a community to design focused interventions [28]. However, studies from Africa and Ethiopia using TB notification data aggregated at larger areas and populations, to assess the spatial distribution of the disease have poor resolution. Moreover, several of the previous studies conducted at broader geographic level did not exclude cases who came from neighbouring areas diagnosed and treated in the reporting health facilities and could have underestimated or overestimated the true disease burden [29,30].

A previous study from Ethiopia reported the spatial and spatio-temporal analysis of TB at kebele level, the smallest administration unit in Ethiopia [27]. However, even smaller geographic units, such as enumeration area (EA) could provide better evidence about the disease distribution pattern at neighbourhoods, and could reduce ecological bias. Investigating spatial variations of disease at coarser geographic scale conceals local and individual variations. A small-area scale analysis is recommended to reduce ecological biases because the analysis is closer to the level of the individual [31]. EA-level aggregation and analysis of the data requires individual TB cases’ location (geographic coordinates) because TB notification data do not have EA-level address of patients. Moreover, an assessment of the relationship between predictors and outcome variables in different geographic levels is important to understand the magnitude and direction of factors contributing to spatial variations of the disease.

To our knowledge, no previous studies from Ethiopia analysed possible disease clustering using the EA and spatial heterogeneities in relationships between PTB CNRs and potential predictors such as physical access, population density, household population size and altitude. A study from southern Ethiopia employed a linear regression to assess a relationship between TB CNRs and selected environmental factors, but the study did not assess spatial heterogeneities in relationships between predictors and TB CNRs [25]. Common analysis such as linear regression models and Ordinary Least Square analysis are often employed to assess the relationships of outcome and predictor variables. However, these analyses do not show heterogeneities in relationships in explanatory variables in each geographic level. Spatial heterogeneity or non-stationarity in relationship between predictors and TB CNRs can be addressed using Geographically Weighted Regression (GWR) which is being used to understand the spatial heterogeneities of other communicable diseases [32–34]. Understanding the spatial distribution of PTB at smallest geographic units helps in devising focused interventions at community level. We therefore aimed to assess the trends in spatial distribution of the disease using the spatial information of individual PTB patients aggregated at EA level. We also looked for spatial heterogeneities in the relationship of disease distribution with access to TB control facilities over 10 years in the Dale district and Yirga Alem town, southern Ethiopia.

## Methods

### Study area and setting

The study was carried out in the Sidama administration, southern Ethiopia. Currently, the administration has a population of about 4.6 million people, 30 districts, 6 towns and 1 city administration. We conducted the study in the Dale district and Yirga Alem town. The Dale district consists of 36 rural and two semi-urban kebeles, and the Yirga Alem town has five urban kebeles. Kebele is the lowest administrative unit in Ethiopia with an average population of about 5000 people. However, most kebeles have the population ranging from about 5000 to 10,000 people. In 2012, the population of the Dale district was 283,424 [35]. We included all 43 kebeles in the study. There are 458 enumeration areas (EAs) in the Dale district and Yirga Alem town, and there are 4–17 EAs in each kebele.

### Data collection and statistical analyses

The data were collected from September 2012 to March 2013. First, we registered PTB cases who were enrolled for treatment from 2003–2012 in all health facilities providing Directly Observed Treatment Short course (DOTS) strategy in the Dale district and Yirga Alem town administration from unit TB registers. We also linked the address of PTB cases that were from the study area but enrolled for treatment in the neighbouring districts to their actual home address and included them in the study [23]. PTB cases from other districts enrolled for treatment in the Dale district and Yirga Alem town were excluded from the study. This was done to avoid under- or over-reporting of the cases in actual administrative areas. Under- or over-reporting of the cases happen because cases from neighbouring areas are not a subset of the total population used as a denominator to calculate CNRs. On the other hand, cases that go to other areas for diagnosis and treatment are often reported outside of the catchment population. These cases are part of the denominator but not included in the numerator to calculate the CNRs and contribute to under reporting in the actual geographic area or in true address [23]. Therefore, overlooking such information in spatial analyses can result in erroneous conclusion.

A list of PTB cases diagnosed and registered for treatment for each urban and rural kebele was prepared. We went to each kebele to verify whether the cases were living in the particular kebele, and the community leaders and elders who were permanent residents of those kebeles were asked to verify the address of PTB cases. We used a contact person for the treatment from the unit TB registers to identify cases that were not identified by the community leaders and elders. Thus, we were able to identify PTB patients who were not identified by the community leaders and elders by interviewing the treatment contacts.

We recruited local guides from the community who knew the residences of the cases in each kebele, and the guides were used to locate the address of the cases. The data collectors were university graduates and trained on the data collection formats (checklist), and how to collect geographic information (latitude, longitude, and altitude) of households and TB control facilities using the geographic positioning system (GPS) tools. We used Garmin GPS-72 H and 76-AM receivers with accuracy less than 10 metres (ranged from 3 metres to 9 metres). A pretested structured questionnaire was used to collect the socio-demographic information and the GPS receivers were used to collect the geographic coordinates and altitude of TB cases’ locations. Altitude data were collected to assess relationships between PTB CNRs because previous studies reported the association between TB CNRs and altitude [19–21]. We obtained informed consent from the study participants prior to the interview and the data collectors interviewed the cases at their residences if they were alive at the time of interview and family members of the cases were interviewed if the cases were deceased. We supervised the data collection process on a daily basis during the study period.

**Figure 1.**
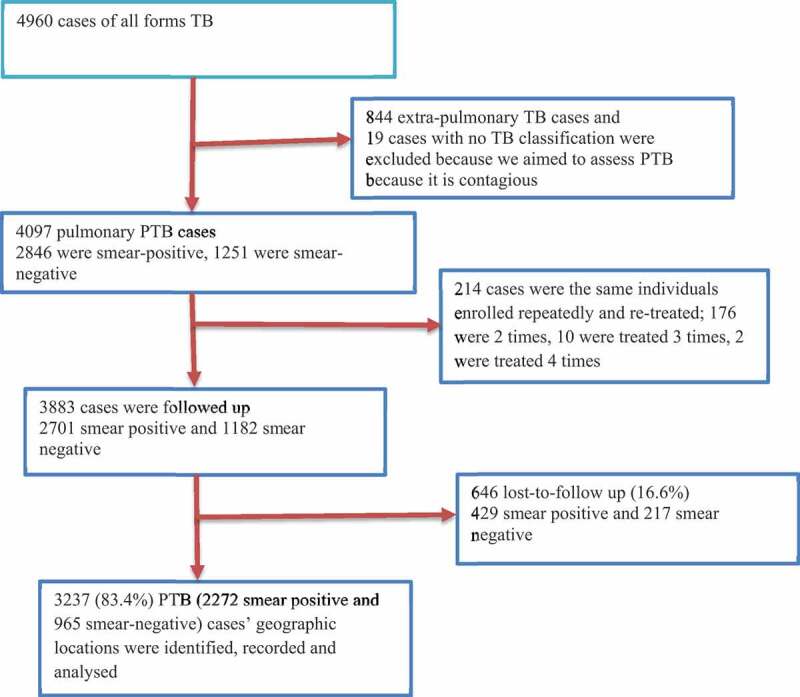
Cohort flow chart of pulmonary TB cases included in the spatial analysis in the Dale district and Yirga Alem town, Ethiopia (2003–2012).

**Figure 2.**
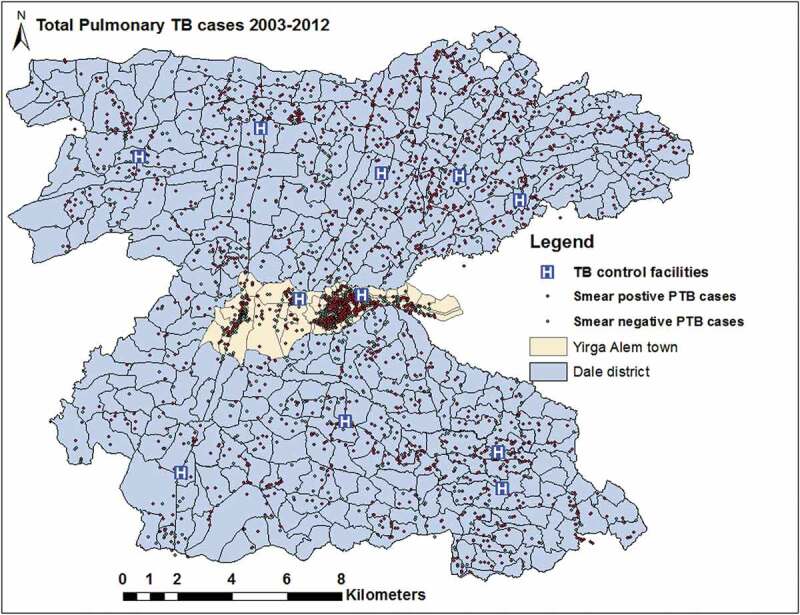
Spatial distribution of individual PTB cases and TB control facilities in the Dale district and Yirga Alem town, Sidama, Ethiopia.

The geographic data (geographic coordinates) were downloaded from GPS receivers using Garmin DNR 5.4.1, 2001 Minnesota, and exported to ArcGIS 10.4. The collected data were double entered by different individuals and errors such as duplication and missing information were corrected. We used a geographic projection of the World Geodetic System (WGS 1984), Universal Transverse Mercator (UTM) Zone 37 N. All PTB cases were geocoded and matched to the kebele and EA-level layers of polygon and points using ArcGIS 10.4 (Figures 1 and 2).

We aggregated the number of cases (point data) in each EA in each kebele for each year for spatial analysis. Enumeration area centroids were used to represent a geographically weighted central location as co-ordinates.

We carried out a hotspot analysis for each year to explore the pattern of spatial clusters and location of PTB cases over 10 years. The hotspot analysis was done by employing the Getis-Ord (Gi*) [36] statistics in ArcGIS 10.4. We used the mean and each year’s CNRs of PTB per 100,000 people for the analysis. The Gi* statistics perform the spatial analysis by looking at each feature within the context of a neighbouring feature. When the local sum of PTB CNR is significantly different from the expected sum, and the difference is too large to be the result of random chance, a statistically significant Z-score results and the computed values of Gi*≥ 1.96 and a P-value of < 0.05 were both considered to represent significant hotspots [36]. Last, we explored the point distributions of individual PTB cases and compared the distribution pattern of them with the results of Gi* statistics.

We also used a Kulldroff’s scan statistics (SaTScan 9.2) [37], to identify purely spatial and space-time clusters. The scan statistics carry out a purely spatial- and space-time cluster analysis, detect clusters size and locations, compute the relative risk and provide a P-value using Monte Carlo simulation. The inputs used were coordinates (EA centroids), population of each EA, and number of cases aggregated at each EA location for spatial and space-time analysis. The number of permutation was set to 999, and a P-value <0.05 considered to be statistically significant high rates. We also included distance to TB control facilities as co-variate. The details of the spatial and space-time analysis are presented elsewhere [37,38].

In 2010, a community-based active case finding campaign was employed by the administration’s health office to improve case detection in the district and the town, and since 2011 a community-based active case finding intervention has been implemented in all kebeles in the study area to improve TB CNRs and treatment outcomes. Moreover, the number of DOTS and TB diagnostic facilities also increased in 2010–2012, which improved physical accessibility to TB services.

We obtained a population size of each kebele and EAs for each year (2003–2012) from the Central Statistical Agency of Ethiopia [35], which was projected from 2007 census. Population density per square kilometre for each EA and for each year was also computed. The elevations above sea level (altitude) for each EA were extracted from ASTER Global Digital Elevation Model Version 2 [39]. We computed mean household population size for each EA. The mean Euclidean distance from each enumeration location to the nearest TB diagnostic and treatment facilities was also computed for each year using proximity-near function of ArcGIS 10.4.

OLS and GWR models are often used to analyse spatial relationship between outcome and explanatory variables. Global regression analysis such as OLS regression models estimate one coefficient for each explanatory variable, averaged for all areas, whereas GWR models estimate a regression coefficient for each location which show spatial heterogeneities in the association between explanatory variables and outcomes. The GWR models also help test a hypothesis that whether the relationships between explanatory and outcome variables vary across space (different geographic areas). The application and details of GWR in epidemiological studies were explained elsewhere [32,34].

The equation used for OLS is; βo +β_1_ +β_2_ + β*_3_ *+β4 +ε, where, (βo +β (*population density*) +β (*physical access*) +β (*Household population size)*+ β (*Altitude*)+ε; whereas the equation for GWRisyi =β 0$\left(ui,vi\right)+\overset{k}{\underset{j=1}{\sum }}\beta j(ui,vi)x$_ij_+εi; where *y*_i_ is the value of the outcome variable (*PTB CNRs*) at the coordinate location *i(EA)* where *ui,vi* represents the coordinates of *i, β*_0_ and *βj* represents the local estimated intercept and effect of variable *j* for location *i*, respectively. The locations near to *i* have a more influence in the estimation of *βj* (*ui,vi*) than locations farther from *i*. In the GWR model localized parameter estimates can be obtained for any location *i* which in turn allows for the creation of a map of parameter values and an examination of the spatial variability (non-stationarity) of these parameters.

First, we carried out the global method, OLS model to look for the relationship between outcome and predictor variables. Then we compared the results of OLS regression model with local GWR model. Corrected Akaike Information Criterion (AICc) is often used to compare the relative fitness of the model and the goodness of fit of the model was also explored using *R-*squared and adjusted *R*-squared. AICc is a measure of model performance and is helpful for comparing other regression models. If a difference in the AICc values between OLS and GWR models vary by more than three [40], a model with the lower AICc is considered as the better fit model.

## Results

### Socio-demographic and clinical characteristics of PTB cases

From 3883 PTB cases geocoded to the actual address, we collected the socio-demographic information and geographic coordinates of 3237(83.4%) PTB cases (Figures 1–2). Twenty smear-negative PTB and 8 smear-positive PTB cases’ locations were from the same households (having the same coordinates), from the same family corresponding to the same geographic location. Fifty-two percent, 1695 cases, were men and 1542 (47.6%) were women. The mean age of the respondents was 30.4 years (SD = 15.4). Majority, 74% of cases were from rural areas (Table 1). About 39% of cases had no schooling and 2272 (70.2%) were smear-positive PTB cases. High proportions of PTB cases were notified in 2011 and 2012. The overall treatment success was 82.3% (increased from 81.6% in 2003 to 90.3% in 2011, and declined to 85.3% in 2012). The death rate was 5.2% during 2003–2012, declined from 11.8% in 2003 to 3% in 2012. (Supplementary Table 1).

**Table 1.** Sociodemographic characteristics of PTB cases in the Dale district and Yirga Alem town in Sidama, southern Ethiopia (2003–2012).

Variables	Frequency (%)
Age group	
0–14	312 (9.6)
15–24	906 (28.0)
25–34	919 (28.4)
35–44	471 (14.6)
45–54	331 (10.2)
55–64	172 (4.3)
≥65	126 (3.9)
**Sex**	
Women	1542 (47.6)
Men	1695 (52.4)
**Place of residence**	
Urban	838 (25.9)
Rural	2399 (74.1)
**Educational Status**	
Primary (1–6)	995 (30.7)
Junior secondary (7–8)	426 (13.2)
High school (9–12)	473 (14.6)
Above high school (12+)	95 (2.9)
No schooling	1248 (38.6)
**TB classification**	
Smear Positive	2272 (70.2)
Smear Negative	965 (29.8)
**Year of treatment**	
2003	255 (7.9)
2004	214 (6.6)
2005	233 (7.2)
2006	207(6.4)
2007	356 (11.0)
2008	370 (11.4)
2009	324 (10.0)
2010	315 (9.7)
2011	451 (13.9)
2012	495 (15.3)
Not mentioned	17 (0.5)
**Treatment outcome**	
Cured	1683 (52)
Completed	981 (30.3)
Died	169 (5.2)
Lost-follow-up	183 (5.7)
Transferred out	103 (3.2)
Treatment failure	5 (0.2)
Unknown	113 (3.5)

### Spatial distribution of pulmonary tuberculosis, access to TB control facilities and environmental factors

**Figure 3.**
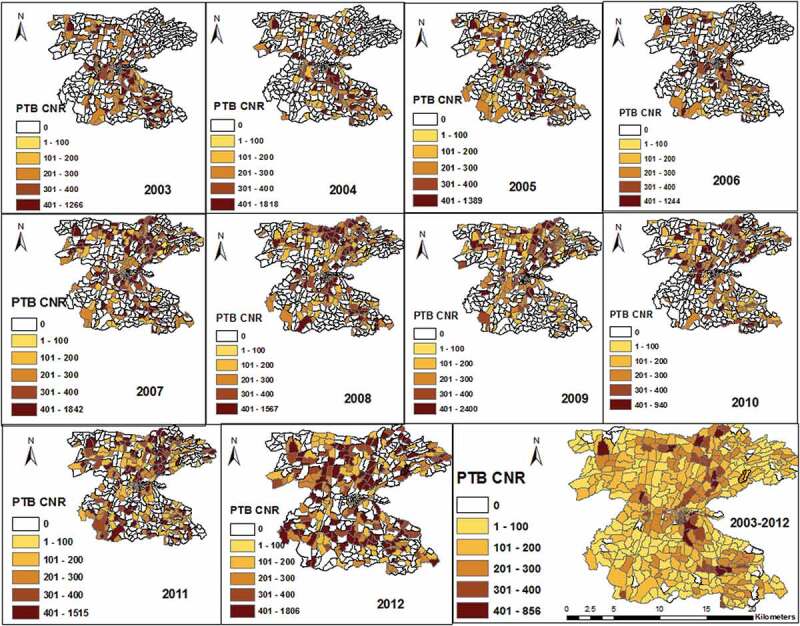
Trends in PTB CNRs between 2003–2012 at EA levels in the Dale district and Yirga Alem town, Sidama, Ethiopia.

The PTB rates varied between kebeles within the district, and ranged from 0/10^5^ to 584/10^5^ people in 2003 and were between 0/10^5^ to 689/10^5^ people in 2012 (Supplementary Figure 2). The mean PTB CNRs between kebeles ranged from 51/10^5^ to 337/10^5^ people from 2003 to 2012. The EA-level mapping also shows a varying pattern of disease distribution in different neighbourhoods. The mean PTB CNRs varied between EAs within kebeles ranged from 0/10^5^ people to 856/10^5^ people from 2003–2012. High rates of PTB was observed in the enumeration areas within Semen Mesenkala, Chume, Moto, Wayicho, Shoye, Tula, Yirgalem town, Manche, and in the Sasamo Dela kebeles in 2003–2012 (Figure 3 and Supplementary Figure 2).

There were also areas with stable and low CNRs over ten years. EAs with new cases of PTB in the following year were adjacent to areas with PTB cases in the preceding years (Figure 3 and Supplementary Figure 3). High PTB CNRs were observed in south-eastern border and central parts around urban setting of the study area in 2003–2005.

The distribution pattern of PTB CNRs changed in 2007–2010. Thus, the high rates of PTB were identified in central and northern parts of the study area. In 2011–2012, high rates were found in northern and southern parts of the study area. Overall high CNRs were identified in northern, central and south-eastern parts of the district (Figure 3 and Supplementary Figure 2).

The mean population density ranged from 8 to 13285 people/km^2^ in EAs. North-eastern, south-eastern and central urban settings have a higher population densities than northern, western and south-western parts of the study area (Supplementary Figure 4).

The mean distance ranged from <1 km to 10 km between EAs in 2003–2008 and declined to <1 km to 6 km in 2012 (the reduction ranged from <1 km to 7.7 km between EAs). North-eastern, western, south-western and north-western areas and borders of the study area have poorer physical access (longer distance) than northern, north-central and south-eastern parts. The mean household population size also varies between EAs (ranged from 1 to 6 people per household). The EAs have altitudes range from 1615 metres above sea level to 2395 metres above sea level, and 92.4% of the EAs have altitudes ranging from 1701 metres to 1999 metres (Supplementary Figure 4).

### Trends of spatial clustering of pulmonary tuberculosis

**Figure 4.**
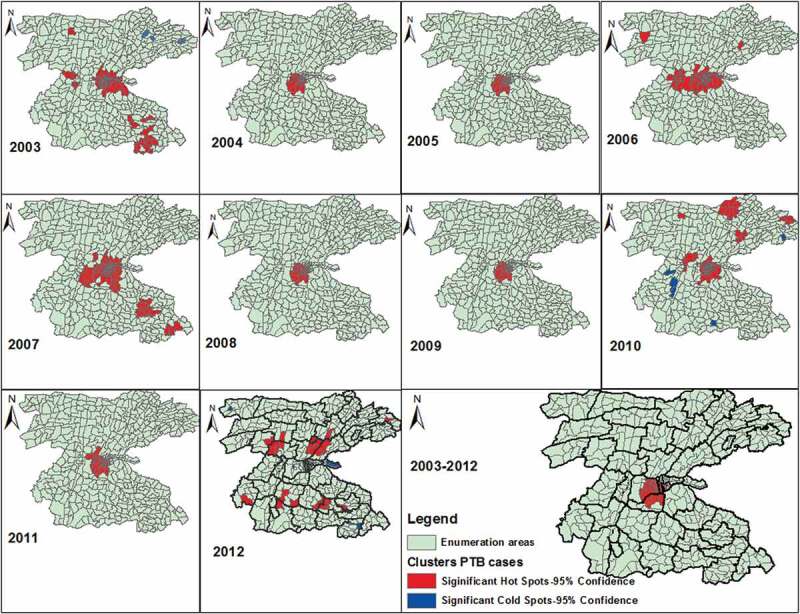
Trends in spatial clustering of PTB between 2003–2012 in the Dale district and Yirga Alem town, Sidama, Ethiopia.

The Gi* statistics identified local clusters (hotspots) of the disease across different areas in different years. Overall the hotspots were identified in 29 (6.3%) of EAs in one rural (Awada), and three urban kebeles (Stadium, Mehal Ketema and Masincho Mewucha), which are located in the central part of the study area (Figure 4). These areas are parts of urban setting and EAs in neighbouring kebeles. No significant disease cluster was detected in Wuha Limat and Kidist Mariam kebels though these kebeles are urban settings with high rates. In other kebeles where the disease clusters were detected, there were also EAs without significant clusters.

**Figure 5.**
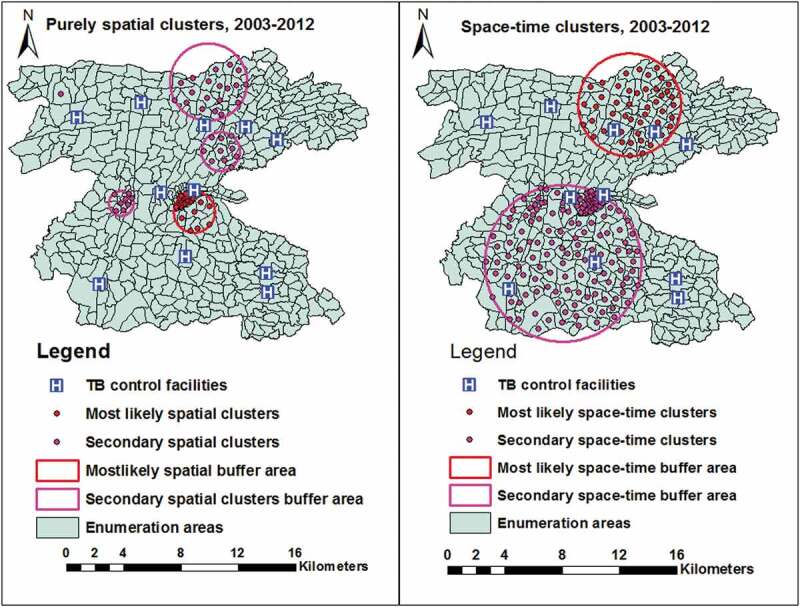
Purely spatial and space-time clusters of PTB in the Dale districts and Yirga Alem town in Sidama, Ethiopia, 20,103–2012.

The purely spatial analysis also showed a most likely clusters in 26 (5.8%) locations and four secondary clusters in in 36 (7.9%) locations in central, western, and northern parts of the study area. The most likely space-time clusters were also identified in 48 (10.5%) locations in northern and north-eastern parts in 2007–2012, and the secondary space-time clusters were identified in central and southern and south-western parts in 2003–2007 (Figure 5 and Supplementary Table 2).

**Table 2.** Summary of OLS results.

Coefficients of OLS results								
Variable	Coefficient	Standard error	t-statistic	Probability	Robust SE	Robust-t	Robust Probability	VIF*
Intercept	308.32	109.87	2.81	0.00523	87.076	3.541	0.00045	
Distance	−17.65	3.11	−5.67	0.0000*	2.83	−6.23	0.0000*	1.18
House hold population	9.073	6.12	1.48	0.139	5.73	1.584	0.1140	1.03
Mean population density	0.015	0.0024	5.93	0.0000*	0.0031	4.797	0.000003*	1.17
Altitude	−0.091	0.057	−1.587	0.1133	0.0443	−2.053	0.04070	1.10
Model diagnostics of OLS								
Number of observation	458							
Multiple R squared	0.20976							
Adjusted R-squared	0.20278							
AICc	5607							
Joint F statistic	30.1	prob (>F), (4453) degrees of freedom					p <0.001	-
Joint Wald statistic	91.15	prob (>chi-squared), (4) degrees of freedom					p <0.001	-
Koenker (BP) statistic	13.45	prob (> chi-squared), (4) degrees of freedom					P = 0.009	-
Jarque-Beta statistic	256.1	Pro (>chi-squared), (2) degrees of freedom					p <0.001	-

Coefficient represents the strength and type of relationship between each explanatory variable and dependent variable

VIF* variance inflation factor

We looked for the pattern of the clusters in each year and found variations in the location of clusters. The EAs with hotspot of the disease ranged from 82 (17%) in 2003 to 28(6.1%) in 2012. In 2003, significant clusters were identified in central location, south-eastern border, and in two EAs in northern part of the district. This pattern has changed and the clusters were identified almost in the same locations and became stable in 2004, 2005, 2008, 2009 and 2011. In 2007, the spatial clusters showed similar pattern with the clusters in 2003 except differences in few locations. In 2010, the spatial clusters identified in 66 (14%) of EAs in new locations in the northern and north-eastern part of the study area. In 2012, the spatial hotspots showed different pattern and were detected in new areas in central and south-central parts of the study area. In the same year, the clusters detected in urban settings in the preceding years were detected no longer (Figure 4).

### Factors associated with the geographical distribution of PTB

The global OLS regression showed a spatially significant relationship between physical access (coefficient −17.7, P <0.0001), population density (0.015, P <0.0001) and altitude (−0.091, p = 0.0407) with PTB CNRs. Household population size did not show a significant association. Overall the model explained 20% of spatial variations (AICc = 5607, R^2^ = 0.2098, adjusted R^2^ = 0.20278) (Table 2).

We employed GWR analysis to determine the spatial heterogeneity in association between access to TB control facilities, population density, population size and altitude, and PTB CNRs. However, we excluded house hold population size because it was not significantly associated with PTB CNRs in the global OLS model. Similarly, we excluded altitude from the GWR model because of local multicollinearity. Distance from TB control facilities and population density were included in the final model.

**Figure 6.**
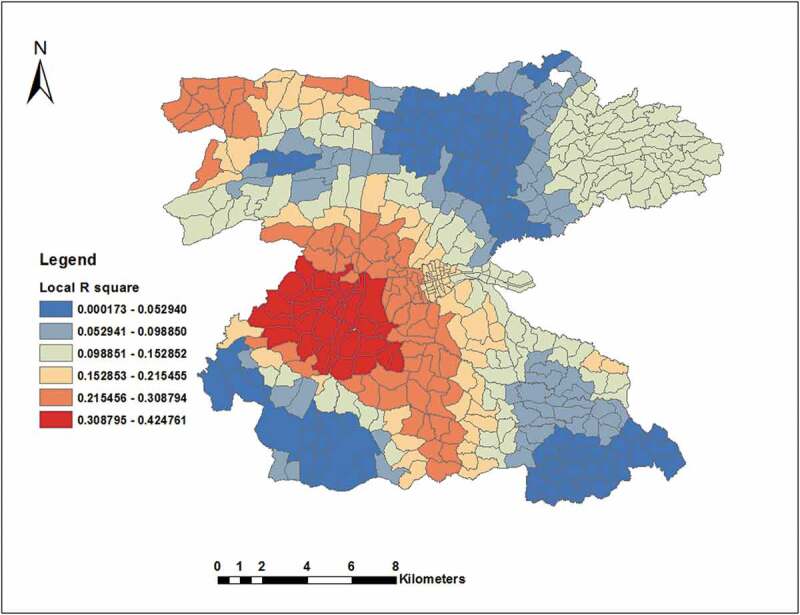
Spatial distribution of local R^2^ values for GWR analysis in the Dale district and Yirga Alem town, Sidama, southern Ethiopia.

Overall the model explained 27% of total model variations (ranging from 0.0002–0.4248 between EAs). The AICc (5591) was less than that for global model and the co-efficient of determination (R^2^ = 0.3359, adjusted R^2^ = 0.2671) was also higher than that of the global model. Therefore, the GWR model was better at modelling the data (Table 3 and Figure 6). We also looked for the distribution of local R^2^ and it was distributed heterogeneously in 458 EAs. The model weakly fitted in northern, north-eastern, south-eastern and north-eastern borders of the study area. The model was better fitted in central and north-western borders of the study area (R^2^ ranging from 0.3204 to 0.4248) (Figure 6).

**Table 3.** Summary results of geographically weighted regression (GWR).

Parameters	Time periods		
	2003–2012	2003–3008	2010–2012
Band width	3016.7	3605.8	3603.4
Residual squares	4558373.6	6719167.6	9537021
Effective number	43	30	33.9
Sigma	104	125.4	149.9
AICc	5587	5587	5908.5
R^2^	0.33587	0.33587	0.18694
Adjusted R^2^	0.26707	0.26707	0.12378

**Figure 7.**
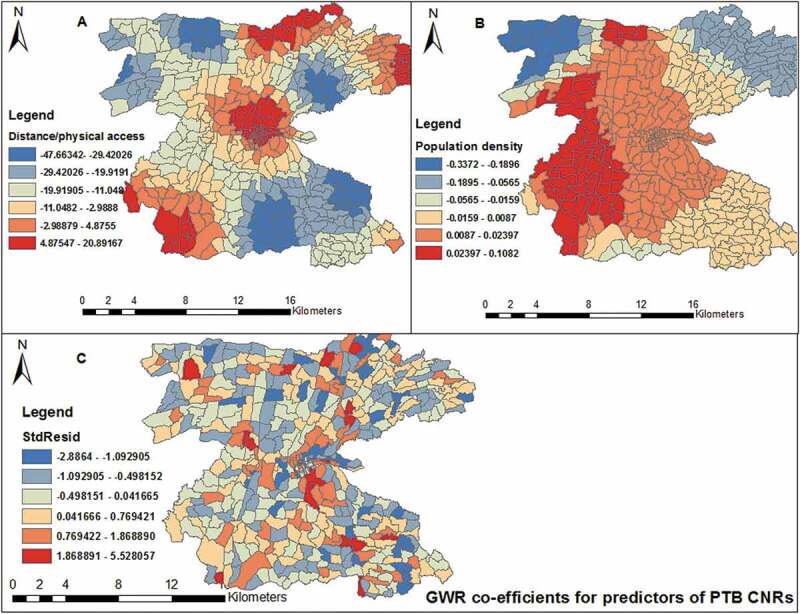
Spatial distribution of local regression coefficients (A) distance, (B) population density, (C) standard residuals for each enumeration area, based on the computation of Geographically Weighted Regression model. The dependent variable was PTB CNRs in Dale district and Yirga Alem town, Sidama, 2003–2012.

We also analysed the data in two time periods (2003–2008 and 2010–2012) to assess whether changes occurred in relationship between physical access and PTB CNRs. This is because in 2003–2008, the physical access to TB control facilities was the same and there were only five TB control facilities in the district. In 2010–2012, physical access to TB control facilities improved due to higher coverage of health facilities (increased to 11 TB control facilities) than in 2003–2008 and due to community-based interventions. Therefore, we found the geographical heterogeneities in the relationship between distance to TB control facilities and TB CNRs in 2003–2008 (AICc = 4745, R^2^ = 0.3088, adjusted 0.2608). In 2010–2012, heterogeneity in the relationship between distance and population density with PTB CNRs was weaker (AICc = 5908, R^2^ = 0.1869, adjusted R^2^ = 0.1238). The model explained only 12% of variations (Table 3).

Distance had negative regression coefficients in south-eastern, north-western and eastern parts of the study area. On the contrary, south-western, central, northern and north-eastern borders of the study area had positive regression coefficients. Thus, areas with longer distance to TB control facilities (with poor access) had lower case notifications and there were also areas with short distance (with better physical access) with low PTB CNRs. Population density also showed similar trends (Figure 7). This implies that factors associated with PTB CNRs were important determinants in some areas and were not important in others. This helps to assess how the relationships between physical accessibility vary spatially with PTB CNRs even after adjusting for population density.

## Discussion

We found varying trends in the distribution of PTB clustering over a decade in the study area. There were also spatial heterogeneities in relationship between access to TB control facilities and population density, and PTB CNRs. As access to TB control facilities improved, the location where spatial clusters occurred has changed. Thus, improved access to TB control facilities could be a contributing factor to a higher case notification of the disease and underlying clusters in most areas. However, the effect of physical access on PTB CNRs changed after coverage of TB control facilities was improved, and during the introduction of community-based interventions in 2010–2012.The introduction of active case finding intervention, which was implemented in all areas might have increased TB care seeking and service utilization. High population density also associated with high PTB CNRs in some areas; however, there were areas with high population density had low PTB CNRs. This implies variations in relationships between population density and PTB CNRs between EAs.

Our finding is in agreement with other studies that reported the occurrence of spatial variations and clustering of TB [5,6]. Possible reasons for the disease clusters in our finding could be partly explained by a varying distribution of access to TB control facilities and differentials in the programme performance. The spatial heterogeneity of the disease distribution could be attributed to variations in socioeconomic status, environmental factors, population density and access to TB control facilities [41].

We also found variations in the distribution pattern of the disease from year to year and the clusters were detected in the neighbourhoods close to areas where the disease clusters were identified in the preceding years. However, the spatial clusters were stable in central part of the district in 2004, 2005, 2008, 2009 and 2011. There were also significant clusters of the disease in the closest neighbourhoods and it was only detected in some EAs within kebeles (not in all EAs). This could partly explain the ongoing transmission of the disease from the neighbourhoods with the highest rates of the disease over years or an improved access to TB diagnostic facilities, which increases TB case notification.

Areas characterized by the disease clusters had a better access to TB control services were urban and semi-urban settings, and were areas with a high population density compared to most areas with low case notifications. However, there were areas with a better access to TB diagnostic facilities and had a high population density, which were not characterized by the highest rates of the disease. The GWR analysis also supported this evidence that there were areas with better access but with low PTB CNRs and others with poor access but with high rates of the disease. This could be due to a disproportionate distribution of risk factors such as HIV infection, factors that increase a contact with infectious cases, poor socioeconomic conditions, and varying performance of TB programs, which could contribute to variations in the burden of the disease over the years [16,17]. We were not able to compare the distribution of HIV and socioeconomic factors with the disease clusters because the data were not available at EA level. A study from China [41] included population density, socioeconomic status, and availability of infectious diseases network reporting agencies in GWR model and identified local variations in smear- positive TB and the spatial heterogeneities in relationship between predictors and smear-positive TB occurrence. The GWR model better explained the variations compared to our study. This could be due to that fact that the authors included socio-economic information in the analysis unlike our study [41]. We therefore suggest further investigation in the future including socioeconomic factors in spatial analysis of the disease to improve our understanding of the spatial heterogeneity and clustering of the disease. On the other hand, neighbourhoods of the areas with the disease clusters in the preceding years might have better awareness and health care seeking about the disease, which could increase the CNRs.

In 2010 a community-based active finding campaign was conducted in the district aiming at improving TB case notification. The campaign might have increased TB case notification in some kebeles. The pattern of spatial clusters of the diseases also has changed and identified in new areas. This could be partly explained by the intervention campaign increased access to information in addition to the increased number of TB control facilities (6 more health centres were functional), which increased physical access.

In 2011, the community-based active case finding intervention was implemented in all kebeles of the district and access to TB control facilities also improved [23,42]. As a result, the CNRs of PTB increased in all kebeles. However, the disease clusters were identified in the same areas where the clusters were detected in the preceding years (2007–2009). In the same year, the disease clusters persisted in urban setting and neighbouring kebeles. These areas had persistent clusters in all years and further investigation and sub-survey should be conducted in these areas in order to identify factors contributing to the high burden of the disease. In 2012, one year after the community-based intervention, the pattern and locations of significant hotspots were changed and we identified EAs with the disease clustering in different kebeles and EAs from the preceding years (2003–2011).

This change in the spatial distribution of the disease could be attributed to the community-based intervention and improved access to TB control services [23,42]. Thus, improved access to TB control services and the community-based intervention might have influenced TB case notifications in some areas, which is reflected by increased PTB CNRs or reduced the transmission of the disease by reducing infectious cases in previously high burden areas. Our finding is consistent with other studies that report improving TB control efforts could change the geographic distribution of the disease [27,43]. The effect of physical access on the PTB CNRs varies year after year due to variations in access to TB control facilities as evidenced in 2010–2012. The GWR model better explained during 2003–2009 than in 2010–2012. This could be partly explained by a change in access to TB control facilities between two time periods.

In our data, even after controlling for physical access in scan statistics, we identified purely spatial and space-time clusters of PTB in the same areas except differences in the number of cluster locations. In GWR analysis, improved access to TB control services was also associated with increased CNRs of the disease, and the number of locations for the spatial clusters was also changed. However, there were areas with improved physical access to TB control facilities with low PTB CNRs. These findings could explain, at least in part, that improved access to TB control facilities might be one of possible reasons for the highest rates of the disease in most areas. Nonetheless, we could not conclude that the highest rates of the disease were attributed to access alone because various socio-demographic and individual risk factors [13,17–19] could contribute to the disproportionate burden of TB.

Evidence from other studies suggests that different isolates of TB could be clustered [44], and the presence of clusters of similar strain of *Mycobacterium tuberculosis* in neighbourhoods indicate an ongoing transmission of the disease. In Ethiopia, different genotypes of *Mycobacterium tuberculosis* were reported [45]. Unfortunately, information on the spatial distribution of different strains of TB is non-existent in southern Ethiopia. Further study is therefore suggested on the spatial distribution of the strains of the *Mycobacterium* including drug resistant TB. We also suggest further studies to identify risk factors explaining the variations including HIV and MDR-TB burden. The application of spatial and temporal analysis to TB control programmes can provide important evidence for identifying clusters of the disease in place and time. This helps policy and decision makers to devise tailored disease control interventions such as improving access to TB control services, resource allocation, and contact tracing. Moreover, integrating the application of GIS with existing health information system of National TB control programmes and other diseases of public health importance could improve disease control and surveillance.

The strengths our study are; the spatial analyses were carried out based on individual TB cases’ data aggregated at EAs level, which have shown better information about the disease clustering within kebeles and neighbourhoods. The spatial modelling (GWR) we used provided better information about the geographic heterogeneities in spatial relationships between explanatory variables and PTB CNRs. The limitations of our study are; we could not include all variables and risk factors because of unavailability of such data. Thus, unmeasured environmental, socioeconomic, and demographic variables might have affected the relationships between PTB CNRs in different geographic areas. Furthermore, we could not confirm the strains of Mycobacterium to report whether the neighbourhood clusters might be attributed to the transmission of the disease with identical strains of *Mycobacterium tuberculosis*. However, the study provided valuable information to understand the pattern of disease clustering and spatial heterogeneities, which could help in devising targeted interventions at local level.

## Conclusion

We found the spatial variations and local clusters of PTB in the study area. Improving access to TB control facilities and programme performance could change the spatial clusters of the disease. We also found spatial heterogeneities in relationships between physical access and population density at EAs, and PTB CNRs. Targeted TB control interventions could be designed in areas where the disease clusters were identified and in the neighbourhoods close to the cluster areas as well as improving access to TB control facilities. Further studies are suggested on the spatial distribution of different strains of *Mycobacterium tuberculosis*, HIV burden including socio-economic factors so as to strengthen the disease surveillance. Understanding spatially varying relationships in explanatory variables and TB burden could help policy makers to devise targeted interventions.

## Supplementary Material

Supplemental Material

## Paper context

Studies from Ethiopia used TB data aggregated at broader geographic levels. No previous studies analysed possible disease clustering using locations of individual TB cases, and spatial variations in relationship between PTB CNRs and potential predictors in small areas. The study found variations in the pattern of spatial clusters of the disease over years, and identified heterogeneities in relationship between TB CNRs and explanatory variables. The findings of the study helps policy and decision makers in targeting tailored interventions at community level.
