# Supplementary material for: Identifying geographical heterogeneity of pulmonary tuberculosis in southern Ethiopia: a method to identify clustering for targeted interventions
Source: Glob Health Action. 2020 Aug 4;13(1):1785737. doi: 10.1080/16549716.2020.1785737 (PMC7480636; doi:10.1080/16549716.2020.1785737)
Supplement: Supplemental Material [file ZGHA_A_1785737_SM8809.zip › Supplemenatry Table 1.docx]

Supplementary Table 1. Treatment outcomes of PTB cases enrolled in Dale district and Yirga Alem town in Sidama, 2003-2012

| Year of treatment | Treatment outcomes | | | | | | | |
| --- | --- | --- | --- | --- | --- | --- | --- | --- |
|  | Completed  N (%) | Cured  N (%) | Died  N (%) | Lost-to-follow up N (%) | Transferred out  N (%) | Treatment failure | Unknown  N (%) | Total  N (%) |
| 2003 | 75 (29.4) | 133 (52.2) | 30 (11.8) | 9 (3.5) | 7(2.7) | 0 | 1 (5.9) | 255 |
| 2004 | 68(31.8) | 113( 52.8) | 17(7.9) | 10(4.7) | 6(2.8) | 0 | 0 | 214 |
| 2005 | 77(33) | 129 (55.4) | 14 (6.0) | 6(2.6) | 6(2.6) | 1 | 0 | 233 |
| 2006 | 104 (50.2) | 80(38.6) | 13(6.3) | 6(2.9) | 3(1.4) | 0 | 1 | 207 |
| 2007 | 134(37.7) | 177(49.9) | 22(6.2) | 17(4.8) | 3(0.8) | 2 (0.6) | 0 | 355 |
| 2008 | 90(24.2) | 202(54.3) | 20(5.4) | 41(11) | 8(2.2) | 0 | 3 | 372 |
| 2009 | 93 (28.8) | 136 (42.1) | 16(5.0) | 29 (9.0) | 25(7.7) | 0 | 23 | 323 |
| 2010 | 113(35.9) | 111(35.2) | 7 (2.2) | 42(13.3) | 33(10.5) | 1 | 6 | 315 |
| 2011 | 82(18.2) | 325(72.1) | 15(3.3) | 14 (3.1) | 4 (0.4) | 0 | 10 | 451 |
| 2012 | 145 (29.3) | 277 (56.0) | 15 (3) | 8(1.6) | 8(1.6) | 1 | 37 | 495 |
| Not-mentioned | 0 | 0 | 0 | 1 | 0 | 0 | 16 | 17 |
