# Supplementary material for: Identifying geographical heterogeneity of pulmonary tuberculosis in southern Ethiopia: a method to identify clustering for targeted interventions
Source: Glob Health Action. 2020 Aug 4;13(1):1785737. doi: 10.1080/16549716.2020.1785737 (PMC7480636; doi:10.1080/16549716.2020.1785737)
Supplement: Supplemental Material [file ZGHA_A_1785737_SM8809.zip › Supplementary Table 2.docx]

Table 2. Purely spatial and space-time clusters of PTB detected by scan statistics in Dale district and Yirga Alem town, Sidama, 2003-2012

| Clusters detected | Number of locations | Number of cases | Year | Expected cases | Likelihood ratio | Relative risk | P- value | Radius |
| --- | --- | --- | --- | --- | --- | --- | --- | --- |
| Most likely | 26 | 456 | 2003-2012 | 280 | 52.0 | 1.74 | <0.001 | 1446 |
| Secondary 1 | 1 | 28 | 2003-2012 | 6 | 22.4 | 4.95 | <0.001 | 0 |
| Secondary 2 | 19 | 212 | 2003-2012 | 135 | 19.8 | 1.61 | <0.001 | 2701 |
| Secondary 3 | 8 | 83 | 2003-2012 | 42 | 15.6 | 1.99 | <0.001 | 864 |
| Secondary 4 | 10 | 107 | 2003-2012 | 65 | 11.5 | 1.67 | 0.006 | 1353 |
| **Space-time clusters of PTB detected by Scan statistics, 2003-2012** | | | | | | | | |
| Clusters Detected | Number of locations | Number of cases | Time frame | Expected cases | Likelihood ratio | Relative risk | P- value | Radius |
| Most likely clusters | 48 | 385 | 2007-2011 | 199 | 74.4 | 2.07 | <0.001 | 3585 |
| Secondary clusters | 152 | 616 | 2003-2007 | 440 | 37.01 | 1.5 | <0.001 | 5489 |
