# Supplementary figures and images for: Identifying geographical heterogeneity of pulmonary tuberculosis in southern Ethiopia: a method to identify clustering for targeted interventions
Source: Glob Health Action. 2020 Aug 4;13(1):1785737. doi: 10.1080/16549716.2020.1785737 (PMC7480636; doi:10.1080/16549716.2020.1785737)

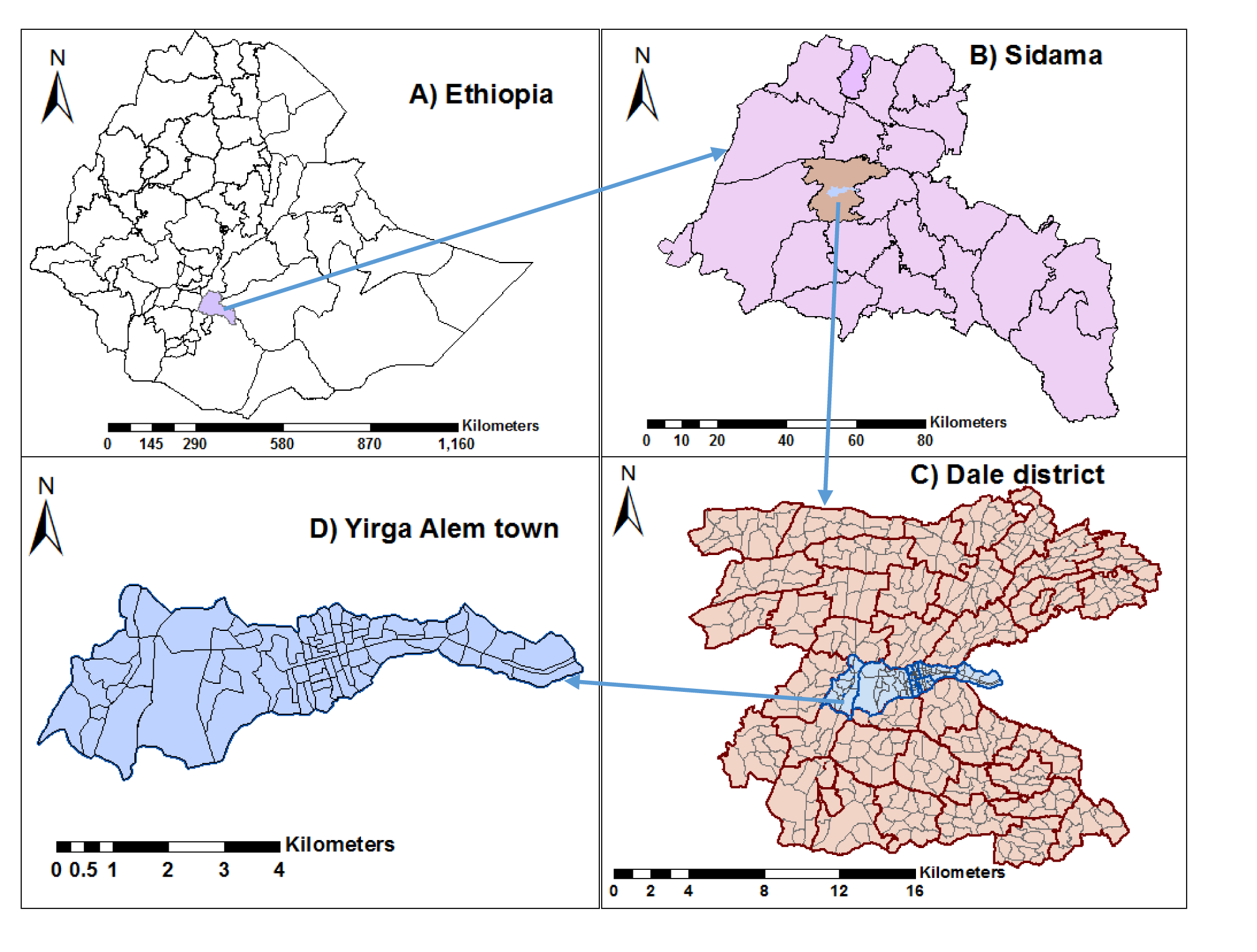

Supplement: Supplemental Material [file ZGHA_A_1785737_SM8809.zip › Supplementary Figure 1.tif]

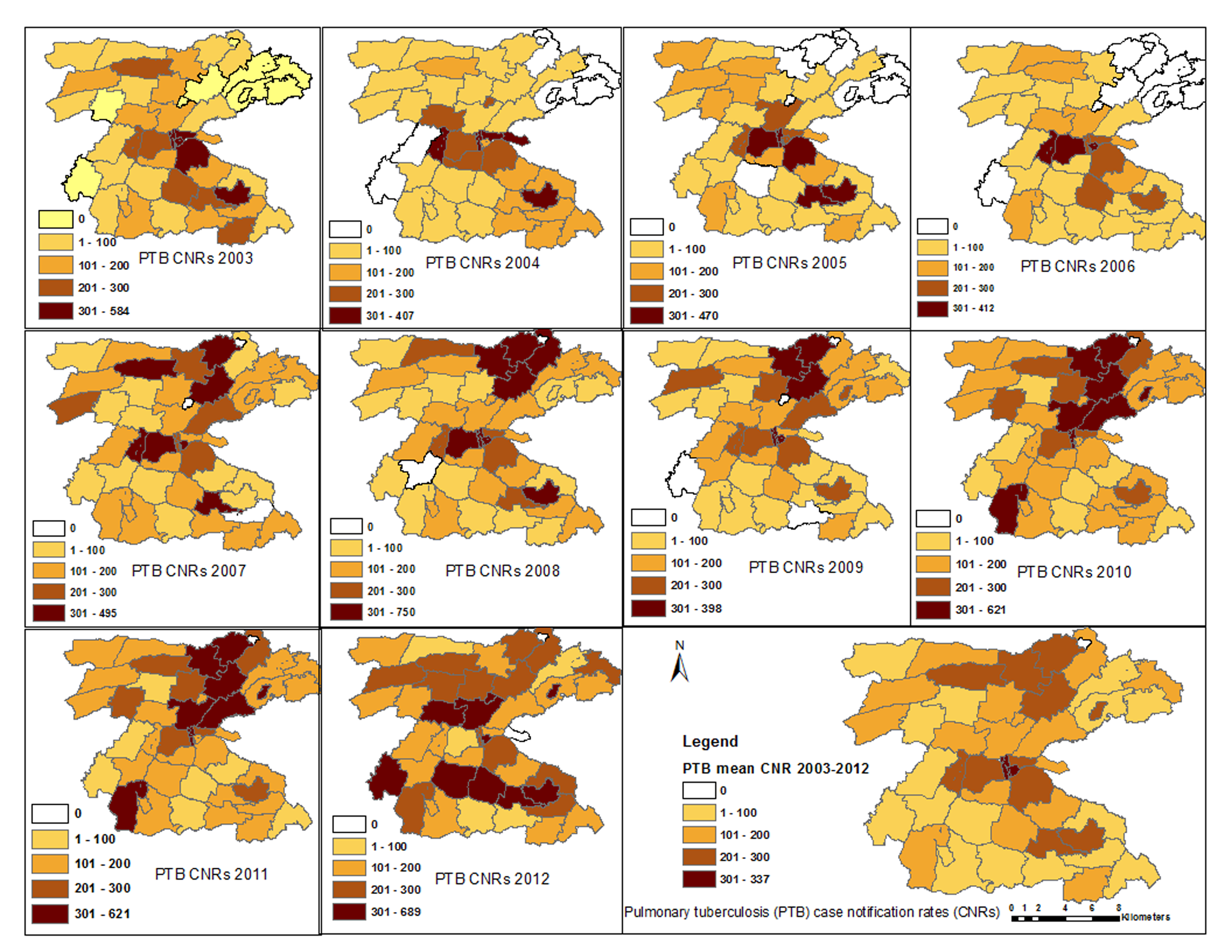

Supplement: Supplemental Material [file ZGHA_A_1785737_SM8809.zip › Supplementary Figure 2.tif]

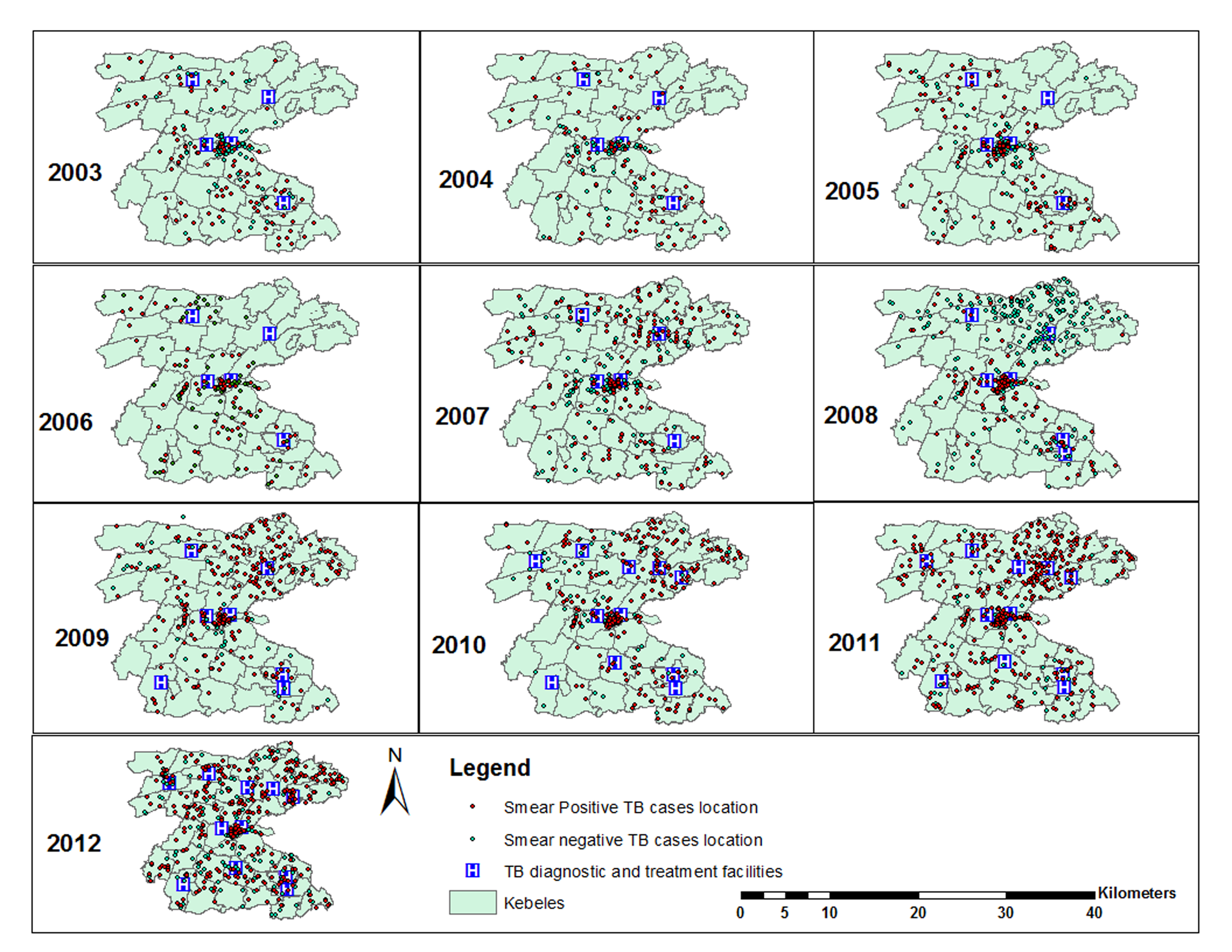

Supplement: Supplemental Material [file ZGHA_A_1785737_SM8809.zip › Supplementary Figure 3.tif]

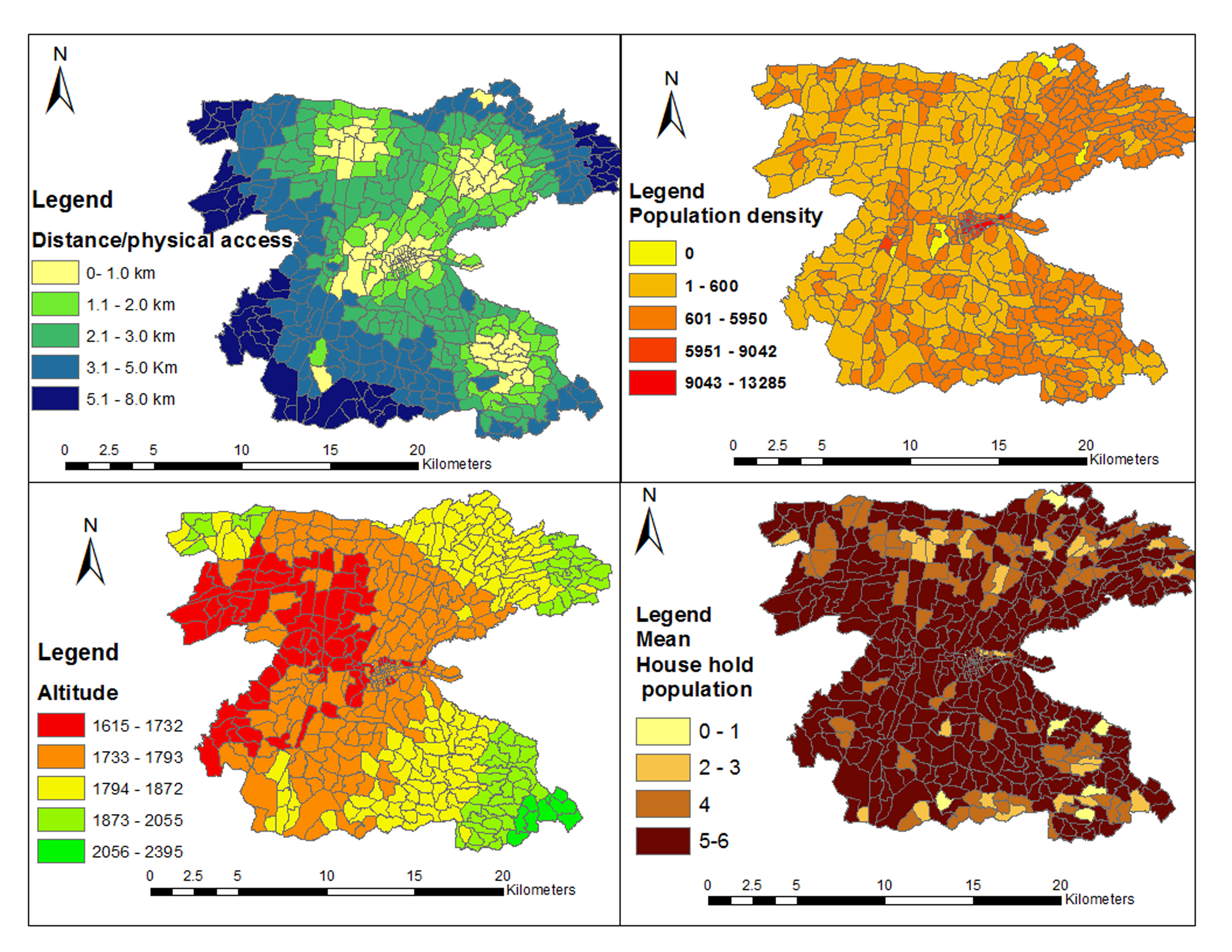

Supplement: Supplemental Material [file ZGHA_A_1785737_SM8809.zip › Supplementary Figure 4.tif]
